# In vitro effects of febrifugine on *Schistosoma mansoni* adult worms

**DOI:** 10.1186/s41182-020-00230-x

**Published:** 2020-06-04

**Authors:** Yoshinori Mitsui, Mitsumasa Miura, Kentaro Kato

**Affiliations:** 1 Department of Parasitology, Institute of Tropical Medicine, Nagasaki University, 1-12-4 Sakamoto, Nagasaki, 852-8523 Japan; 2 Department of Eco-epidemiology, Institute of Tropical Medicine, Nagasaki University, 1-12-4 Sakamoto, Nagasaki, 852-8523 Japan

**Keywords:** Febrifugine, *Dichroa febrifuga*, Antischistosomal drug, *Schistosoma mansoni*, Antimalarial drug

## Abstract

**Background:**

Reports on the antischistosomal effect of several antimalarial drugs such as artesunate, mefloquine, and amodiaquine suggest that febrifugine, which exerts an antimalarial effect, can also be expected to possess antischistosomal potential. The present study investigates the antischistosomal effects of febrifugine.

**Methods:**

In experiment 1, *Schistosoma mansoni* adult worm pairs were incubated in a medium alone as a control or supplemented with febrifugine at 0.05, 0.1, 0.2, and 0.5 μg/ml for 14 days. The morphology of the worms and the egg production of the female worms were observed simultaneously. In experiment 2, the incubation was conducted as in experiment 1, except that the febrifugine concentrations were reduced to 0.005, 0.01, and 0.02 μg/ml. In addition, *S*. *mansoni* adult worms were incubated with either 0.5 μg/ml febrifugine or none as a control for 5 days and stained with neutral red dye.

**Results:**

Febrifugine significantly reduced the survival of *S*. *mansoni* male and female worms at concentrations of 0.02–0.5 μg/ml following incubation for 14 days and remarkably inhibited the daily egg output of the female worms. The non-treated male and female worms remained morphologically normal within the period of 14 days, whereas male and female worms treated with febrifugine at different concentrations gradually twisted and subsequently died. In addition, *S*. *mansoni* adult worms were incubated with either 0.5 μg/ml febrifugine or none as a control for 5 days and stained with neutral red dye. Non-treated male worms were morphologically normal and stained dark red with neutral red, while febrifugine-treated male worms appeared similar to those in the control group and were stained at a slightly lower level of dark red than the non-treated male worms. Non-treated female worms were morphologically normal, and their intestinal tract and vitellaria were stained deep red and dark red, respectively. In contrast, febrifugine-treated female worms were morphologically damaged, and their intestinal tract and vitellaria remained mostly unstained and stained dark red, respectively.

**Conclusion:**

Febrifugine exerts potent antischistosomal effects and can be expected to contribute to the development of a novel antischistosomal drug.

## Background

Schistosomiasis persists as a serious parasitic disease in 78 countries in both tropical and subtropical areas around the world [1]. Some 61 million people were treated with praziquantel (PZQ) by 2014, but an estimated 260 million people continue to require treatment for schistosomiasis. Chemotherapy with PZQ is the mainstay of strategies to control schistosomiasis [2], but the extensive reliance on PZQ raises concerns about the emergence of drug-resistant parasites [3, 4]. Therefore, the development of an antischistosomal drug in addition to or as an alternative to PZQ is urgently needed.

Artemisinin derivatives such as artemether and artesunate (ART) are well-known antimalarial drugs that exert an antischistosomal effect [5, 6]. Other antimalarial drugs in current use, such as mefloquine (MQ), amodiaquine (AQ), and primaquine (PQ), also display antischistosomal effects [7, 8], further suggesting that compounds possessing antimalarial activity may display antischistosomal potential.

Febrifugine (Feb) was isolated from the roots and leaves of *Dichroa febrifuga* Lour [9, 10], a plant used for centuries in China to treat malarial fevers [11]. Subsequently, Feb was also isolated from the leaves of certain *Hydrangea* species [12]. Henderson et al. (1949) demonstrated the antimalarial effect of Feb against *Plasmodium lophurae* infection in ducks, *P*. *relictum* in canaries, and *P*. *cynomolgi* in rhesus monkeys in experimental studies [13]. Furthermore, Hewitt et al. (1952) reported that Feb was approximately 100 times more effective than quinine (QN) in counteracting *P*. *lophurae* infection in ducks [14]. Feb was on the verge of serving as a potent new antimalarial drug, but its severe side effects precluded its use as a clinical antimalarial drug for half a century [13, 15]. In recent years, a derivative of Feb called halofuginone has attracted keen attention because of its wide range of beneficial biological activities, encompassing malaria and cancer as well as fibrosis-related and autoimmune diseases [16]. At the same time, Feb has come to light as a leading compound for the development of new antimalarial drugs [17, 18]. Feb is a quinazolinone alkaloid with a basic chemical structure different from that of the artemisinin derivatives and quinoline alkaloids observed in current antimalarial drugs such as QN, chloroquine, PQ, AQ, and MQ [19, 20]. Thus, Feb is a promising candidate for the development of a novel antimalarial drug, and, in view of the mechanism of other antimalarial drugs, it may also exert antischistosomal effects. To our knowledge, however, the effect of Feb on schistosomes has not been investigated to date. The assessment of the antischistosomal effects of Feb, therefore, constitutes a new approach to the development of a novel antischistosomal drug.

In the present groundbreaking study, we evaluated the in vitro antischistosomal effects of Feb according to the modified method of Mitsui et al. (2009) [6]. The effects of the drug were examined with regard to (1) the egg production of female *Schistosoma mansoni* adult worms, (2) the survival of adult worms, (3) the gross morphological alterations of the adult worms caused by the drug, and (4) the antischistosomal effect of Feb on *S*. *mansoni* adult worms assessed using neutral red (NR) non-fluorescent dye.

## Materials and methods

### Chemicals and media

Feb dihydrochloride was purchased from Sigma-Aldrich Co. (St**.** Louis**,** Missouri**,** USA**)** and diluted in deionized water (DW) to a concentration of 10 mg/ml as a free-base stock solution. This stock Feb solution was then added to medium NCTC 135 (Sigma-Aldrich Co.) containing a 0.5% solution of antibiotics (penicillin 5000 units and streptomycin 5000 mg/l; Gibco**,** Langley, Oklahoma, USA) at concentrations of 0.005–0.5 μg/ml. A NR dye was purchased from Nacalai Tesque, Inc. **(**Kyoto**,** Japan) and dissolved in DW at a concentration of 0.1**%** as a stock solution (pH 3.1).

### Parasite strain

A Puerto Rican strain of *S*. *mansoni* (NIH-Sm-PR-1) was routinely maintained by passage through 6-week-old female ICR mice (Japan SLC**,** Inc**.** Hamamatsu**,** Japan**)** and *Biomphalaria glabrata* snails (Newton’s NIH Puerto Rican/Brazilian M-line) in the Laboratory Animal Centre. At 8 weeks after infection with 200 *S*. *mansoni* cercariae, adult worms were collected by the perfusion technique as previously described by Smithers and Terry [21] and washed twice with NCTC 135 medium.

### Incubation of *Schistosoma mansoni* adult worm pairs with febrifugine

The incubation was conducted as previously described by Mitsui et al. (2009) [6], except for differences in the test drug, the drug concentration, and replacement of media over a period of 14 days. Before exposure to Feb, each pair of *S*. *mansoni* adult worms was preincubated for 1 day with 0.5 ml of NCTC 135 medium in a single well of a 24-well multi-well plate (Sumitomo Bakelite Co. Ltd, Osaka, Japan) in a 5% CO_2_ incubator at 37 °C. After preincubation, the adult worms were used for subsequent experiments.

In experiment 1, 30 male/female S. mansoni adult worm pairs were randomly assigned to five groups of six pairs each. Then, each worm pair was transferred into a well containing 0.5 ml of NCTC 135 medium supplemented with 0 as control, 0.05, 0.1, 0.2, or 0.5 μg/ml Feb. The plates were continuously incubated in a 5% CO2 incubator at 37 °C. The medium alone or medium supplemented with the drug was changed every 2 to 3 days for 14 days. The number of eggs produced daily was counted, and the morphological appearance of the worms was observed visually under a Nikon SMZ 800 stereoscopic microscope (Nikon Corporation, Tokyo, Japan). Assessment of the “dead or alive” status of adult worms was determined by the movement of the worm upon stimulation with a needle. Worms were classified as “dead” when they failed to respond to the needle stimulation. Images of worms treated with the drug were obtained using a digital Senamal C-mount camera (Microscope Network, Saitama, Japan) mounted on the stereoscopic microscope.

The procedure in experiment 2 was identical to that of experiment 1, except for the concentration of Feb. That is, 24 adult worm pairs were randomly assigned to four groups of six worm pairs each, and each pair was continuously incubated in the medium supplemented with 0 as control, 0.005, 0.01, or 0.02 μg/ml Feb for 14 days with replacement of the medium every 2 to 3 days.

### Staining of febrifugine-treated *Schistosoma mansoni* adult worms with neutral red

The antischistosomal effect of Feb on *S*. *mansoni* adult worms was assessed with NR dye according to a modified method previously described by Mitsui and Kato (2018) [22]. The male and female adult worms were randomly allocated into two groups of three worms each: control and 0.5 μg/ml Feb groups. Each group was incubated for 1 day with 2 ml of NCTC 135 medium alone in a 35 mm plastic dish (Sumitomo Bakelite Co. Ltd) in a 5% CO_2_ incubator at 37 °C. Subsequently, three male and female worms from each group were transferred to a new plastic dish containing 2 ml of the medium alone as a control or supplemented with 0.5 μg/ml Feb. The dishes were continuously incubated in a 5% CO_2_ incubator at 37 °C for 5 days with one change of the medium. Then, 40 μl of the stock NR solution was added to each dish (final concentration: 0.002%) and incubated in a 5% CO_2_ incubator at 37 °C for 1 ho. Then, the living male and female worms were individually placed in 20 μl of NETC 135 medium mounted on a regular microscope slide (76 × 26 × 0.9–1.2 mm; Matsunami Glass Industries, Ltd, Osaka, Japan) and sandwiched with a coverslip (18 × 18 mm; Matsunami Glass Industries, Ltd). Then, the coverslip was sealed with a commercial fingernail polish. The images of the worms were captured using an Olympus BX43 microscope (Olympus Corporation, Tokyo, Japan) equipped with a digital camera (Sony Alpha NEX-5, Sony Corporation, Tokyo, Japan).

### Statistical analysis

Data were analyzed using Epi-Info software (Centers for Disease Control and Prevention, Atlanta, GA, USA). The daily egg output per female adult worm was expressed as the arithmetic mean (± standard error). The Kruskal-Wallis or Mann-Whitney *U* test was used to compare daily egg outputs between the control and Feb-treated groups. The median survival time of *S*. *mansoni* adult worms was calculated. Comparison of worm survival time between the control and Feb-treated groups and between male and female worms in the Feb-treated groups was performed using the log-rank test.

## Results

### Effect of febrifugine on the egg production of *Schistosoma mansoni* adult worm pairs

The effect of Feb on the daily egg output of female adult worms is shown in Table 1. In experiment 1, the mean daily egg output during the 1-day preincubation period was not significantly different among the control and 0.05, 0.1, 0.2, and 0.5 μg/ml Feb-treated groups (*P* = 0.88 for all comparisons). During the first 2 days of incubation, the mean daily egg output in the 0.05–0.5 μg/ml Feb-treated groups was not significantly lower than that in the control group, but on the third day of incubation, the mean daily egg output in the 0.5 μg/ml Feb-treated group was significantly lower than that in the control group (*P* < 0.01). Furthermore, on the fourth day of incubation, the mean daily egg output in the 0.05, 0.1, and 0.2 μg/ml Feb-treated groups was significantly lower than that in the control group (*P* < 0.05, *P* < 0.01, and *P* < 0.01), and on the fifth day, the mean daily egg output in the Feb-treated groups was zero or almost zero.

**Table 1:** Inhibitory effect of febrifugine on the daily egg output of six paired *Schistosoma mansoni* adult worms within 7 days

Febrifugine concentration (μg/ml)	Mean daily egg output (± standard error)							
	Day 0	Day 1	Day 2	Day 3	Day 4	Day 5	Day 6	Day 7
Experiment 1								
0 (control)	30.0 ± 9.9	115.3 ± 16.3	111.2 ± 18.2	52.8 ± 14.3	47.2 ± 17.6	36.5 ± 12.9	7.3 ± 4.2	5.7 ± 3.3
0.05	20.2 ± 5.8	102.2 ± 13.8	81.0 ± 19.8	43.3 ± 3.8	4.8 ± 3.1^a^	0.3 ± 0.3^b^	0	
0.1	19.7 ± 9.2	101.3 ± 16.1	74.5 ± 7.8	35.8 ± 3.0	1.2 ± 0.3^b^	0		
0.2	24.7 ± 10.5	51.5 ± 17.5	68.8 ± 29.6	18.0 ± 9.1	0.8 ± 0.5^b^	0.2 ± 0.2^b^	0	
0.5	18.3 ± 6.0	85.3 ± 20.3	50.7 ± 15.5	4.5 ± 2.5^b^	1.2 ± 0.8^b^	0		
Experiment 2								
0 (control)	26.5 ± 12.6	94.2 ± 18.8	101.2 ± 21.6	98.3 ± 18.9	57.5 ± 14.8	22.0 ± 6.6	8.7 ± 2.4	4.8 ± 1.9
0.005	20.3 ± 6.3	92.0 ± 18.3	69.0 ± 15.1	88.7 ± 29.0	66.2 ± 26.1	13.2 ± 6.1	6.7 ± 2.6	1.7 ± 0.8
0.01	25.3 ± 10.1	86.5 ± 5.9	78.8 ± 15.8	80.0 ± 15.4	48.0 ± 21.3	6.8 ± 5.7	1.7 ± 1.5	0.7 ± 0.4
0.02	16.8 ± 7.2	100.2 ± 28.5	89.5 ± 13.9	84.7 ± 29.8	12.0 ± 5.4^a^	1.7 ± 0.9^a^	0	

Mean daily egg output during the one-day preincubation (day 0) among the control and febrifugine-treated groups was not significantly different in experiment 1 and 2 (Kruskal-Wallis test, *P* = 0.88 and 0.92 for all comparisons, respectively)

^a^*P* < 0.05

^b^*P* < 0.01, compared with the corresponding control group according to a Mann-Whitney *U* test

In experiment 2, the mean daily egg output during the 1-day preincubation period was not significantly different among the control and 0.005, 0.01, and 0.02 μg/ml Feb-treated groups (*P* = 0.85 for all comparisons). During the first 3 days of incubation, the mean daily egg output in the Feb-treated groups did not differ significantly from that in the control group, but on the fourth day of incubation, the mean daily egg output in the 0.02 μg/ml Feb-treated group was significantly lower than that in the control group (*P* < 0.05). On the other hand, during the incubation period of 7 days, the mean daily egg output in the 0.005 and 0.01 μg/ml Feb-treated groups was not significantly lower than that in the control group.

### Effect of febrifugine on the survival time of *Schistosoma mansoni* adult worm pairs

The survival time of *S*. *mansoni* adult worm pairs incubated with 0 (control), 0.05, 0.1, 0.2, and 0.5 μg/ml Feb in experiment 1 and with 0 (control), 0.005, 0.01, and 0.02 μg/ml Feb in experiment 2 is shown in Table 2. None of the worms died in the control groups during the 14-day incubation period in either experiment 1 or 2. On the other hand, although none of the worms died in the 0.005 and 0.01 μg/ml Feb-treated groups, all the male and female worms in the 0.02–0.5 μg/ml Feb-treated groups in experiments 1 and 2 died within the 14-day incubation period. Moreover, a significant difference was observed in the survival time between male and female worms in the 0.1 and 0.2 μg/ml Feb-treated groups.

**Table 2:** Effect of febrifugine on the survival time of six paired *Schistosoma mansoni* adult worms within 14 days

Concentration of febrifugine (μg/ml)	Median survival days (range)		*P* value^b^
	Male	Female	
Experiment 1			
0 (control)	ND	ND	
0.05	12.0 (12–13)^a^	12.0 (11–12)^a^	0.18
0.1	11.0 (11)^a^	9.0 (9–11)^a^	0.00
0.2	10.5 (9–11)^a^	8.5 (8–9)^a^	0.00
0.5	8.0 (7–8)^a^	7.0 (7–8)^a^	0.09
Experiment 2			
0 (control)	ND	ND	–
0.005	ND	ND	–
0.01	ND	ND	–
0.02	13.0 (13–14)^a^	13.5 (13–14)^a^	0.57

A log-rank test was used for the statistical analysis

*ND* death was not observed in any of the worms over the incubation period of 14 days

^a^*P* value < 0.01, compared with the corresponding control group

^b^Compared between male and female

### Gross morphological alterations of *Schistosoma mansoni* adult worms caused by febrifugine

**Fig. 1:**
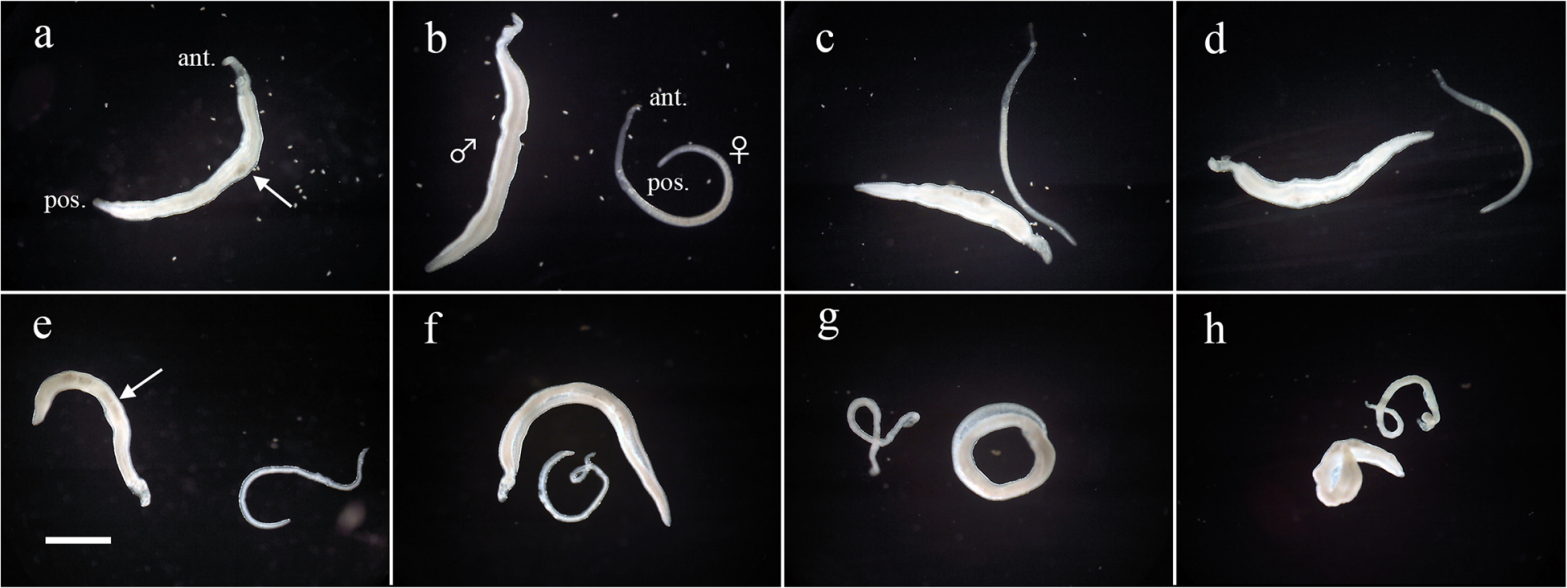
Effect of febrifugine on the gross morphological alteration of *Schistosoma mansoni* adult worms. Paired *S*. *mansoni* adult worms were continuously incubated in media alone for 3 (**a**), 5 (**b**), 7 (**c**), and 8 (**d**) days (control group), while paired adult worms were incubated with 0.5 μg/ml febrifugine for 3 (**e**), 5 (**f**), 7 (**g**), and 8 (**h**) days. Arrows indicate the localization of black contents. The symbols ♂ and ♀ indicate male and female worms, respectively. *ant*. anterior part of body, *pos*. posterior part of body. Scale bar = 1 mm

The morphological appearance of *S*. *mansoni* adult worm pairs untreated or treated with Feb was observed daily under a dissecting microscope. When paired adult worms were consecutively incubated in medium alone (control group) in experiment 1, all the male and female adult worms were still in pairs within three days (Fig. 1a). The black content in the body of male worms mostly disappeared within 3 days (Fig. 1a) and the black content in the body of separated female worms also faded over a period of 5 days (Fig. 1b). In the subsequent incubation, very little further morphological alteration was observed in either male or female worms (Fig. 1c, d). When paired adult worms were treated with 0.5 μg/ml Feb for 3 days, no remarkable morphological alteration was observed in the body of the male worms or the separated female worms (Fig. 1e). However, on day 5 after exposure to Feb, although the male worms appeared similar to those in the control (Fig. 1f), the female worms showed a visible twisting in the anterior part of the body (Fig. 1f). After subsequent incubation, all the male worms showed obvious twisting (Fig. 1g, h), and all the female worms were further twisted (Fig. 1g, h). Similar morphological alterations were observed in male and female worms treated with Feb at the other concentrations of 0.02, 0.05, 0.1, and 0.2 μg/ml during the incubation periods of 10–14, 6–13, 6–11, and 5–11 days, respectively (data not shown).

### Staining of febrifugine-treated *Schistosoma mansoni* adult worms with neutral red dye

**Fig. 2:**
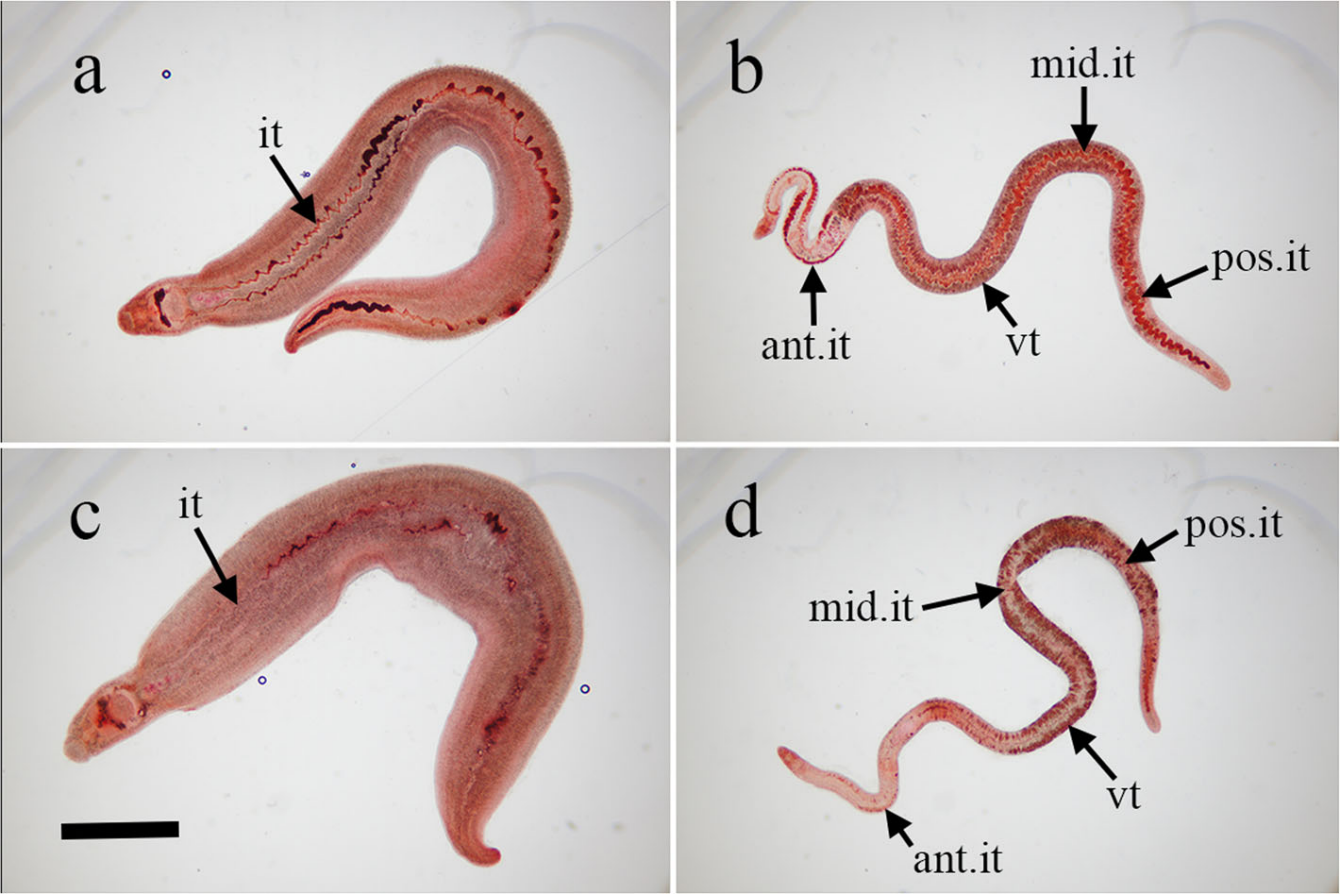
Neutral red staining of *Schistosoma mansoni* adult worms incubated with febrifugine. Male and female *S*. *mansoni* adult worms were incubated with none (male: **a**, female: **b**) or 0.5 μg/ml febrifugine (male: **c**, female: **d**) for 5 days and stained with neutral red. *it* intestinal tract, *vt* vitellaria, *ant*. anterior, *mid*. middle, *pos*. posterior. Scale bar = 1 mm

Within 5 days after male and female *S*. *mansoni* adult worms were treated with none as control or 0.5 μg/ml Feb, the worms were stained with NR. Images of the staining of non- and Feb-treated *S*. *mansoni* adult worms are presented in Fig. 2. When non-treated male and female worms were stained with NR, the whole body of the males appeared dark red in color and the intestinal tract was stained deep red (Fig. 2a). With regard to the body of female worms, the status of NR staining was as follows: anterior intestinal tract, deep red; vitellaria, dark red; middle and posterior intestinal tract, deep red (Fig. 2b). On the other hand, the Feb-treated male worms were stained at a slightly lower shade of dark red than the non-treated male worms, and the accumulation of NR in the intestinal tract was less than that of the non-treated male worms (Fig. 2c). Furthermore, the staining status of Feb-treated female worms was as follows: anterior intestinal tract, slightly stained deep red; vitellaria, dark red; middle and posterior intestinal tract, mostly unstained (Fig. 2d).

## Discussion

Several antimalarial drugs have been reported to exhibit an antischistosomal effect [6–8]. It follows that Feb, which exerts an antimalarial effect, may also possess antischistosomal potential, but the possibility has never been investigated. The present study was conducted to achieve a clear understanding of the antischistosomal effect of Feb.

When pairs of *S*. *mansoni* adult worms were treated with Feb at concentrations of 0.02–0.5 μg/ml, the daily egg output was significantly lower than that of the corresponding control groups after 3 or 4 days of incubation (Table 1), while the daily egg output of the 0.01 μg/ml Feb group was similar to that of the control group. Thus, the minimum concentration of Feb that effectively inhibits the daily egg output of adult worm pairs is presumed to be 0.02 μg/ml. Furthermore, Feb significantly reduced the survival time of all adult worms at concentrations of 0.02–0.5 μg/ml during the 14-day incubation period (Table 2), while no death of worms was observed in the 0.01 μg/ml Feb group, further suggesting that the minimum concentration of Feb effective in inhibiting the survival of adult worm pairs is also 0.02 μg/ml. In addition, the effect of Feb on the daily egg output and survival time of adult worm pairs was dependent on the concentration and duration of exposure to the drug. The significant difference in survival time between male and female worms treated with Feb at concentrations of 0.1 or 0.2 μg/ml (Table 2) suggested that female worms are more susceptible to Feb than male worms. However, since the survival time of female worms was similar to that of male worms at the lower concentration of 0.02 or 0.05 μg/ml, no definite conclusion could be drawn with regard to the difference between female and male worms in susceptibility to Feb.

The non-treated male and female adult worms of *S*. *mansoni* remained morphologically normal within the period of 14 days, while the male and female worms treated with Feb at different concentrations gradually twisted and subsequently died (Fig. 1). The onset of morphological alteration in the Feb-treated worms was apparently dependent on the concentration and duration of exposure to the drug. Furthermore, the morphological alteration caused by Feb obviously occurred earlier in female than in male worms (Fig. 1f).

Subsequently, we applied NR as a non-fluorescent dye to better assess the antischistosomal effect of Feb on *S*. *mansoni* adult worms. A cationic non-fluorescent dye, NR is absorbed by living cells but not by dead or damaged cells. When *S*. *mansoni* adult worms were incubated with either 0.5 μg/ml Feb or none as a control for 5 days and stained with NR, the non-treated male worms were morphologically normal and stained dark red with NR (Fig. 2a), while the Feb-treated male worms appeared similar to those in the control group and were stained at a slightly lower level of dark red than the non-treated male worms (Fig. 2c), suggesting only slight damage caused by Feb. In contrast, the staining status of the 0.5 μg/ml Feb-treated female worms differed remarkably from that of the non-treated female worms (Fig. 2b, d). The non-treated female worms were morphologically normal, and their intestinal tract and vitellaria were stained deep red and dark red, respectively. On the other hand, the Feb-treated female worms were morphologically damaged, and their intestinal tract and vitellaria remained mostly unstained and stained dark red, respectively. The marked difference in staining status between the non- and Feb-treated female worms suggests that Feb inflicts severe damage on female worms, especially the intestinal tract.

The antimalarial effect of halofuginone and other Feb derivatives is due to the inhibition of prolyl-tRNA synthetase (PRS) in the malaria parasite [18, 23]. Moreover, Jain et al. (2017) reported that synthetic derivatives of Feb selectively and potentially inhibit the PRS of not only malaria but also other protozoan parasites such as *Plasmodium*, *Toxoplasma*, *Cryptosporidium*, and *Eimeria* [24]. Hence, the antischistosomal effect of Feb may be attributable to a mode of action similar to that observed in the protozoan parasites. However, no investigation related to the antischistosomal effect of Feb has been conducted to date, and the question remains as to whether other Feb derivatives exert an antischistosomal activity like that shown in the present study. Clearly, further studies are needed to elucidate the mode of action of Feb against *S*. *mansoni* adult worms.

The present study evaluated the antischistosomal effect of Feb on egg production of *S*. *mansoni* female worms and the survivability of male and female worms and their morphological changes. We showed that the minimal concentration of Feb exhibiting antischistosomal activity is 0.02 μg/ml. However, since the present study focused broadly on the effect on *S*. *mansoni* worms as a way to evaluate the antischistosomal activity of Feb, the cytotoxicity thereof remained to be examined. On the other hand, Jiang et al. (2005) and Kikuchi et al. (2014) evaluated the cytotoxicity of Feb against mammalian neuronal cells (NG108), macrophages (J774), and mouse L929 cells, and showed that the half-maximal inhibitory concentrations were 0.064, 0.081, and 0.169 μg/ml, respectively [17, 25]. Thus, the therapeutic range of concentrations at which Feb is effective for treating schistosomiasis with acceptable side effects is presumed to be narrow. However, despite severe side effects [13, 15], the antimalarial potency of Feb has encouraged medicinal chemists to develop new Feb analogues with potent antimalarial activity but negligible toxicity to humans [17, 19]. These analogues may also be expected to exert an antischistosomal effect and to play a role in the treatment of schistosomiasis.

## Conclusion

The present study demonstrated that Feb significantly reduced the survival time of paired *S*. *mansoni* male and female adult worms at concentrations of 0.02–0.5 μg/ml during in vitro cultivation for 14 days and inhibited the egg production by female worms. The antischistosomal effect of Feb is a topic of keen interest and promises to contribute to the development of a novel antischistosomal drug.

## Data Availability

Not applicable.

## Abbreviations

ant.: Anterior or anterior part of body
AQ: Amodiaquine
ART: Artesunate
DW: Deionized water
Feb: Febrifugine
it: Intestinal tract
mid.: Middle
MQ: Mefloquine
NR: Neutral red
pos.: Posterior or posterior part of body
PQ: Primaquine
PRS: Prolyl-tRNA synthetase
PZQ: Praziquantel
QN: Quinine
vt: Vitellaria
WHO: World Health Organization

## References

[1] World Health Organization (WHO) (2016). Schistosomiasis: number of people treated worldwide in 2014. Wkly Epidemiol Rec.

[2] World Health Organization (WHO) (2011). Helminth control in school-age children: a guide for managers of control programmes.

[3] Doenhoff MJ, Kusel JR, Coles GC (2002). Resistance of Schistosoma mansoni to praziquantel: is there a problem?. Trans R Soc Trop Med Hyg.

[4] Wang W, Wang L, Liang YS (2012). Susceptibility or resistance of praziquantel in human schistosomiasis: a review. Parasitol Res.

[5] Lescano SZ, Chieffi PP, Canhassi RR (2004). Antischistosomal activity of artemether in experimental schistosomiasis mansoni. Rev Saude Publica.

[6] Mitsui Y, Miura M, Aoki Y (2009). In vitro effects of artesunate on the survival of worm pairs and egg production of Schistosoma mansoni. J Helminthol.

[7] Keiser J, Chollet J, Xiao SH, Mei JY (2009). Mefloquine—an aminoalcohol with promising antischistosomal properties in mice. PLoS Negl Trop Dis.

[8] Mitsui Y, Aoki Y (2010). In vitro effect of current antimalarial drugs on the survival of paired Schistosoma mansoni adult worms and their egg production. Trop Med Health.

[9] Koepfli JB, Mead JF, Brockman JA (1947). An alkaloid with high antimalarial activity from Dichroa febrifuga. J Am Chem Soc.

[10] Koepfli JB, Mead JF, Brockman JA (1949). Alkaloids of *Dichroa febrifuga*. I. Isolation and degradative studies. J Am Chem Soc.

[11] Jang CS, Fu FY, Huang KC (1948). Pharmacology of ch'ang Shan (Dichroa febrifuga) a Chinese antimalarial herb. Nature..

[12] Ablondi F, Gordon S, Morton J (1952). An antimalarial alkaloid from hydrangea. II. Isolation. J Org Chem.

[13] Henderson FG, Rose CL, Harris PN (1949). Gamma-Dichroine, the antimalarial alkaloid of ch'ang Shan. J Pharmacol Exp Ther.

[14] Hewitt RI, Wallace WS, Gill ER (1952). An antimalarial alkaloid from hydrangea XIII. The effects of various synthetic quinazolones against plasmodium lophurae in ducks. Am J Trop Med Hyg.

[15] Coatney GR, Cooper WC, Culwell WB (1950). Studies in human malaria. XXV. Trial of febrifugine, an alkaloid obtained from Dichroa febrifuga Lour against the Chesson strain of plasmodium vivax. J Natl Malar Soc.

[16] Pines M, Spector I. Halofuginone—the multifaceted molecule. Molecules. 2015;20(1):573-594. doi:10.3390/molecules20010573. 10.3390/molecules20010573 PMC6272571 25569515

[17] Jiang S, Zeng Q, Gettayacamin M (2005). Antimalarial activities and therapeutic properties of febrifugine analogs. Antimicrob Agents Chemother.

[18] Jain V, Yogavel M, Oshima Y (2015). Structure of prolyl-tRNA synthetase-halofuginone complex provides basis for development of drugs against malaria and toxoplasmosis. Structure..

[19] Kikuchi H, Tasaka H, Hirai S (2002). Potent antimalarial febrifugine analogues against the plasmodium malaria parasite. J Med Chem.

[20] Oliveira AB, Dolabela MF, Braga FC (2009). Plant-derived antimalarial agents: new leads and efficient phythomedicines. Part I. alkaloids. An Acad Bras Cienc.

[21] Smithers SR, Terry RJ (1965). The infection of laboratory hosts with cercariae of *Schistosoma mansoni* and the recovery of the adult worms. Parasitology..

[22] Mitsui Y, Kato K (2018). Application of non-fluorescent dyes to assess the antischistosomal effect of antimalarial drugs on *Schistosoma mansoni* adult worms. Jpn J Infect Dis.

[23] Jain V, Kikuchi H, Oshima Y (2014). Structural and functional analysis of the anti-malarial drug target prolyl-tRNA synthetase. J Struct Funct Genom.

[24] Jain V, Yogavel M, Kikuchi H (2017). Targeting prolyl-tRNA synthetase to accelerate drug discovery against malaria, leishmaniasis, toxoplasmosis, cryptosporidiosis, and coccidiosis. Structure.

[25] Kikuchi H, Horoiwa S, Kasahara R, Hariguchi N, Matsumoto M, Oshima Y (2014). Synthesis of febrifugine derivatives and development of an effective and safe tetrahydroquinazoline-type antimalarial. Eur J Med Chem.

